# Rich biotin content in lignocellulose biomass plays the key role in determining cellulosic glutamic acid accumulation by *Corynebacterium glutamicum*

**DOI:** 10.1186/s13068-018-1132-x

**Published:** 2018-05-10

**Authors:** Jingbai Wen, Yanqiu Xiao, Ting Liu, Qiuqiang Gao, Jie Bao

**Affiliations:** State Key Laboratory of Bioreactor Engineering, East China University of Science and Technology, 130 Meilong Road, Shanghai, 200237 China

**Keywords:** Glutamic acid, Lignocellulose, *Corynebacterium glutamicum*, Biotin, Vitamin B

## Abstract

**Background:**

Lignocellulose is one of the most promising alternative feedstocks for glutamic acid production as commodity building block chemical, but the efforts by the dominant industrial fermentation strain *Corynebacterium glutamicum* failed for accumulating glutamic acid using lignocellulose feedstock.

**Results:**

We identified the existence of surprisingly high biotin concentration in corn stover hydrolysate as the determining factor for the failure of glutamic acid accumulation by *Corynebacterium glutamicum*. Under excessive biotin content, induction by penicillin resulted in 41.7 ± 0.1 g/L of glutamic acid with the yield of 0.50 g glutamic acid/g glucose. Our further investigation revealed that corn stover contained 353 ± 16 μg of biotin per kg dry solids, approximately one order of magnitude greater than the biotin in corn grain. Most of the biotin remained stable during the biorefining chain and the rich biotin content in corn stover hydrolysate almost completely blocked the glutamic acid accumulation. This rich biotin existence was found to be a common phenomenon in the wide range of lignocellulose biomass and this may be the key reason why the previous studies failed in cellulosic glutamic acid fermentation from lignocellulose biomass. The extended recording of the complete members of all eight vitamin B compounds in lignocellulose biomass further reveals that the major vitamin B members were also under the high concentration levels even after harsh pretreatment.

**Conclusions:**

The high content of biotin in wide range of lignocellulose biomass feedstocks and the corresponding hydrolysates was discovered and it was found to be the key factor in determining the cellulosic glutamic acid accumulation. The highly reserved biotin and the high content of their other vitamin B compounds in biorefining process might act as the potential nutrients to biorefining fermentations. This study creates a new insight that lignocellulose biorefining not only generates inhibitors, but also keeps nutrients for cellulosic fermentations.

**Electronic supplementary material:**

The online version of this article (10.1186/s13068-018-1132-x) contains supplementary material, which is available to authorized users.

## Background

Glutamic acid is a five-carbon amino acid with the annual production of three million tons as flavor enhancer [1]. Together with the potential of glutamic acid to act as commodity monomer chemicals for productions of polyesters and polyamides, it requires a major shift of feedstock from food crops-derived glucose to non-food carbohydrate alternatives [2]. Among all the available feedstocks options, lignocellulose biomass provides one of the most promising options due to its abundance and availability to produce various valued-added products based on fermentation [3, 4]. *Corynebacterium glutamicum* is the major industrial strain for glutamic acid production since its first isolation at 1960s [5]. It also provides a versatile cell factory to produce multiple bio-based chemicals and biofuels far beyond the traditional l-amino acids [5]. The potential of *C. glutamicum* in lignocellulose biorefinery processes was also well demonstrated [5, 6]. However, very few studies on *C. glutamicum* concerned the fermentation of the most traditional and important glutamic acid using lignocellulose feedstock. The only relevant studies are on the pentose utilization to accumulate low titer of glutamic acid by *C. glutamicum* [7, 8].

We tried to establish a practical glutamic acid fermentation process by *C. glutamicum* using corn stover feedstock by dry acid pretreatment, biodetoxification, and high solids loading saccharification and fermentation, which had successfully applied to produce high titer of ethanol [9], long chain fatty acid [10], lactic acid [11], gluconic acid [4] and citric acid [12]. However, failures on glutamic acid accumulation were encountered during our cellulosic glutamic acid fermentations. Generally, the over-degradation products such as furfural, 5-hydroxymethylfurfural (HMF), acetic acid, and phenolic aldehydes generated from pretreatment step are considered as the major challenges of biorefining saccharification and fermentations [13]. Cellulosic glutamic acid fermentation scenario was significantly different from the general biorefining fermentation cases with much greater cell growth of *C. glutamicum* in inhibitor containing corn stover hydrolysate (CSH) than that in the generally used complex medium, but failed to accumulate target product, glutamic acid.

We noticed that the phenomenon of high cell growth rate and low glutamic acid accumulation of cellulosic glutamic acid fermentation is similar to the general glutamic acid fermentation using glucose under excessive biotin condition [14, 15]. Biotin is a crucial factor to the dominant industrial biotin auxotrophic bacterium *C. glutamicum* for its essential role to act as cofactor for acetyl-CoA carboxylase in fatty acid synthesis [16]. The absence of biotin suppresses the cell growth but the excessive biotin blocks glutamic acid secretion [14, 17], unless certain surfactants such as Tween 60 [18], β-lactam antibiotics such as penicillin [19], or other cell wall inhibitors such as ethambutol [20] are used to disrupt the over-strengthened cell structure and activate glutamic acid secretion as well as redirect the carbon flux to glutamic acid synthesis [21]. Indeed, we identified the existence of surprisingly high contents of biotin in corn stover feedstocks to be the determining factor of cellulosic glutamic acid fermentation and the induction by penicillin for high titer glutamic acid was demonstrated. In addition to its specific function on glutamic acid, biotin also plays important role in other fermentations such as lysine [22] and arginine [23], ethanol [24], and lactic acid [25]. High biotin content was also found in the other generally used lignocellulose biomass such as rice straw, wheat straw, sugarcane bagasse, and *P. communis* reeds, but not in poplar sawdust.

Besides biotin, vitamin B family includes thiamin (vitamin B1), riboflavin (vitamin B2), niacin (vitamin B3), pantothenate (vitamin B5), pyridoxine (vitamin B6), folic acid (vitamin B9), and cobalamin (vitamin B12) consisting important cofactors of metabolisms for nearly all kinds of organisms [26–30]. The contents of all eight vitamin B compounds in the biorefinery process were also recorded and the major vitamin B members maintained at high concentration levels after harsh pretreatment. This study reveals the determining factor of biotin in lignocellulose to cellulosic glutamic acid fermentation, and the existence of rich vitamin B compounds as potential nutrient supplementations to general biorefining fermentations.

## Results and discussion

### Suppressed cellulosic glutamic acid accumulation and the solution to high titer fermentation

**Fig. 1:**
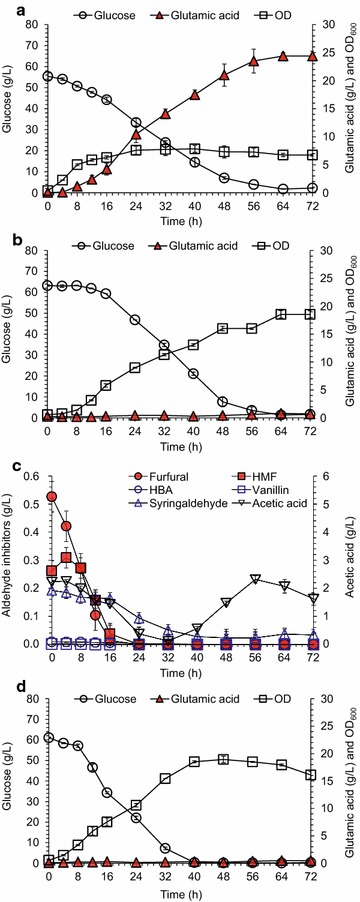
Glutamic acid fermentation of *C. glutamicum* S9114 in the complex medium and CSH. **a** In the biotin-limited complex medium. **b** In the non-detoxified CSH. **c** Inhibitor degradation in the non-detoxified CSH. **d** In the CSH (15% solid content, w/w). Fermentation was carried out in 250 mL flasks containing 30 mL of complex medium or corn stover hydrolysate as described in the Methods section. Mean values were presented with error bars representing at least two standard deviations

The potential of glutamic acid fermentation by the dominant industrial fermenting strain *C. glutamicum* S9114 using corn stover as feedstock was evaluated (Fig. 1). As a control, glutamic acid fermentation was first carried out in the biotin-limited complex medium (containing 0.5 g/L corn steep liquor, CSL) which was considered as a conventional treatment for glutamic acid fermentation [11]. The cells grew quickly and 54.4 ± 1.6 g/L of glucose was converted to 24.4 ± 0.8 g/L of glutamic acid by *C. glutamicum* S9114 within 72 h (Fig. 1a). While in the non-detoxified CSH prepared by hydrolyzing 15% (w/w) of the pretreated corn stover (without inhibitor removal or detoxification), 63.2 ± 0.4 g/L of glucose was completely consumed and the cell growth was almost threefold greater than that in the biotin-limited complex medium, but almost no glutamic acid accumulation was observed (Fig. 1b). *C. glutamicum* exhibited high tolerance to the inhibitors derived from the pretreatment step (Fig. 1c) by quickly converting furfural, HMF, syringaldehyde, 4-hydroxybenzaldehyde (HBA) and vanillin into the corresponding less toxic alcohols or acids. Acetic acid was a byproduct of *C. glutamicum* [31], but the *C. glutamicum* strain showed strong tolerance to it. However, the essential removal of inhibitors from pretreated corn stover feedstock by biodetoxification did not result in the observed accumulation of glutamic acid (Fig. 1d). Nutrients additions of varying dosages of corn steep liquor (CSL) and inorganic salts (KH_2_PO_4_, MgSO_4_, FeSO_4_, and/or MnSO_4_) into the corn stover hydrolysate also did not result in the observable changes of cell growth and glutamic acid accumulation. These results indicate that the inhibitor existence or nutrient deficiency in corn stover hydrolysate is not the determining factor on glutamic acid accumulation by *C. glutamicum*.

**Fig. 2:**
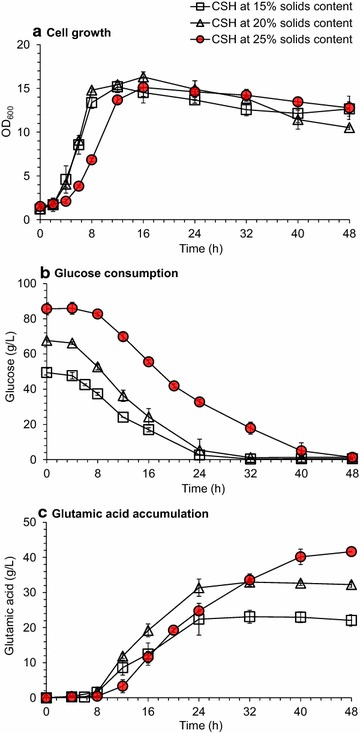
Triggering of glutamic acid secretion from *C. glutamicum* S9114 cells by penicillin induction in CSH. **a** Cell growth. **b** Glucose consumption. **c** Glutamic acid accumulation. CSHs were prepared from the pretreated and biodetoxified corn stover feedstock at 15, 20, and 25% (w/w) solids content, respectively. Fermentation was carried out in a 3-L fermentor as described in the Methods section. Mean values were presented with error bars representing at least two standard deviations

We noticed that the phenomenon of low glutamic acid accumulation with high cell mass formation was frequently observed in the conventional glutamic acid fermentation under the existence of excessive biotin content [14, 15]. A general solution for low glutamic acid accumulation caused by excessive biotin is to use β-lactam antibiotics such as penicillin to trigger glutamic acid secretion by disrupting the crosslinking of peptidoglycan of *C. glutamicum* cell envelope [17, 19]. A significant increase of glutamic acid accumulation by penicillin addition was observed in corn stover hydrolysate (Fig. 2): the cell growth reached OD_600_ of 14–16 at 16 h with only slight lag phase (Fig. 2a), the glucose was completely consumed (Fig. 2b), and glutamic acid was accumulated considerably (Fig. 2c) after penicillin addition at the middle stage of the exponential growth of *C. glutamicum* (OD_600_ at 8–9). The maximum glutamic acid titer reached 41.7 ± 0.1 g/L with the yield of 0.5 g/g within 48 h using the CSH at 25% solids content (w/w). Although corn stover hydrolysate contained glucose, xylose, as well as small amount of arabinose, galactose, and mannose, the present *C. glutamicum* S9114 only utilizes glucose. Thus the yield was calculated only based on the glucose consumption. This meaningful penicillin induced glutamic acid accumulation using lignocellulose as fermentation feedstock marked a potential to compete with the conventional glutamic acid fermentation using the starch derived glucose feedstock (over 120 g/L) [32]. Even the glutamic acid titer here is relative low and the penicillin addition is expensive for industrial application, the overall economic feasibility would be significantly improved by further elevating the bioconversion level, eliminating the use of the expensive penicillin inducer, and applying the innovative multistage fermentation design [33].

### Identification of excessive biotin in corn stover hydrolysate and its crucial role on glutamic acid accumulation

**Fig. 3:**
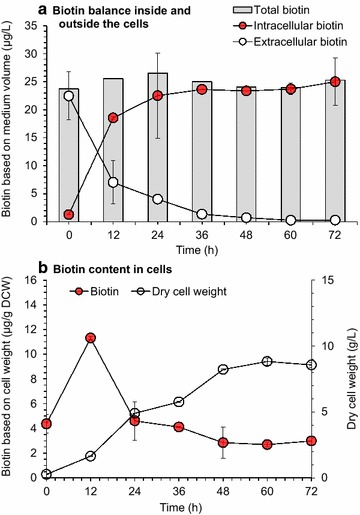
Balance of intracellular and extracellular biotin during glutamic acid fermentation by *C. glutamicum* in CSH. **a** Biotin balance inside and outside the cells. **b** Biotin content in cells. CSH at 15% solids content (w/w) was used here. Fermentation was carried out in flasks as described in the Methods section without penicillin induction. Mean values were presented with error bars representing at least two standard deviations

We designed a series of stepwise experiments for identification of excessive biotin in lignocellulose and its function on glutamic acid fermentation. The first identification of excessive biotin in corn stover hydrolysate was by microbiological assay using VitaFast Kit (R-Biopharm AG, Darmstadt, Germany). The surprisingly high biotin concentration of 22.5 ± 4.3 μg/L was determined in the CSH at 15% solid content (w/w) (Fig. 3) prepared by hydrolyzing 15% (w/w) of the pretreated and biodetoxified corn stover. This biotin level is approximately two orders of magnitude greater than that of the complex medium (0.21 ± 0.02 μg/L), and tenfold greater than the “suboptimal” biotin level (2–5 μg/L) [15] used for glutamic acid fermentation. The excessive biotin in corn stover hydrolysate was quickly transported into the *C. glutamicum* cells with the well conserved mass balance between the extracellular and intracellular biotin (Fig. 3a). Although the intracellular biotin content declined from the maximum of 11.3 ± 0.1 μg/g dry cell weight (DCW) to approximately 2.7 ± 0.2 μg/g DCW with increasing cell mass (Fig. 3b), this is still too high for glutamic acid secretion [15].

**Fig. 4:**
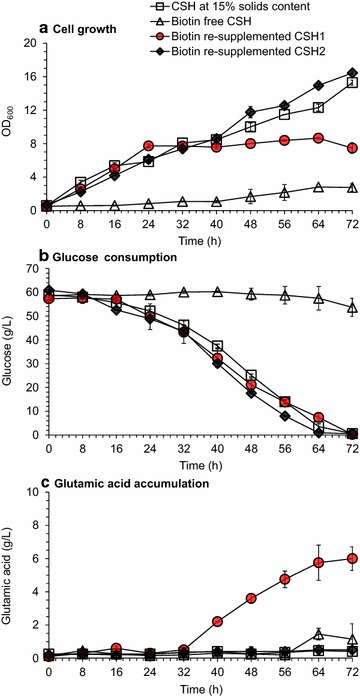
Biotin removal and re-supplementation on cell growth and glutamic acid fermentation of *C. glutamicum* S9114. **a** Cell growth. **b** Glucose consumption. **c** Glutamic acid accumulation. CSH at 15% solids content (w/w) was used here. The initial biotin content of CSH was 22.5 ± 4.3 μg/L. Mean values were presented with error bars representing at least two standard deviations

Then a biotin depletion experiment was designed by selectively depleting biotin from corn stover hydrolysate using a specific biotin binding protein, avidin [34], followed by the re-supplementation of biotin into the hydrolysate using pure biotin (Fig. 4). The depletion of biotin by adding optimal dosage of avidin (20 mg/L, equivalent to 200–300 units/L) led to the significantly depressed cell growth of *C. glutamicum* (Fig. 4a) and the suspended glucose consumption (Fig. 4b). After biotin was re-supplemented by adding 1.0 μg/L of pure biotin, the cell growth and glucose consumption were restored and the glutamic acid accumulated to 6.0 ± 0.7 g/L (Fig. 4c). To further confirm the excessive biotin on glutamic acid accumulation, we added 23 μg/L biotin to the biotin free CSH. As expected, comparable cell growth and glucose consumption with original CSH was observed, but no glutamic acid was accumulated again. The key role of biotin on cell growth and glutamic accumulation in the CSH was fully demonstrated.

The crucial role of excessive biotin in corn stover hydrolysate on glutamic acid accumulation was further identified at the transcriptional level by RT-qPCR analysis (Additional file 1: Table S1). The biotin-limited medium (containing 0.5 g/L CSL) was used as control of the biotin rich CSH (15% solids content, w/w) and the biotin-rich complex medium (containing 5.0 g/L CSL). A similar transcription pattern was observed in the biotin rich CSH and in the biotin rich complex medium. The genes involving phospholipid synthesis including cyclopropane-fatty-acyl-phospholipid synthase gene *ufaA* [35], phosphatidyl-glycerophosphate synthase gene *pgsA1* [35], and phosphatidylinositol α-mannosyltransferase gene *pima* [35] were significantly up-regulated, indicating the cell membrane may strengthened under the condition of excessive biotin. Similarly, the cell wall structure also was seem to be strengthened in the presence of excessive biotin as the genes involving peptidoglycan synthesis including two septum-peptidoglycan synthetase genes *ftsI* [36], two d-alanyl-d-alanine carboxypeptidase genes *dacB* and *dac* [37] were significantly up-regulated. On glutamate synthesis pathway, the genes *odhA* and *sucB* encoding α-ketoglutarate dehydrogenase complex (ODHC) subunits e1o and e2o [38] on the key node of carbon flux network to glutamic acid [39] were obviously up-regulated, while the two glutamate dehydrogenase genes (*gdh*) showed no differential change. Since the carbon flux distribution around the α-ketoglutarate branch was depend more on the ODHC activity [39], the carbon flux to TCA cycle at that node could be enhanced instead of glutamate synthesis. Furthermore, the gene *yggb* (CGS9114_RS01440) encoding putative mechanosensitive channel (MscCG) homolog responsible for glutamate secretion [21] was obviously down-regulated, indicating the glutamic acid secretion would be curtailed. The up-regulation of biotin transporter genes *bioYMN* [40] was in agreement with the experimental observation of the quick biotin uptake by the cells (Fig. 3). The genes responsible for biotin biosynthesis were not examined in this study, because *C. glutamicum* was the naturally biotin auxotrophic strain which cannot synthesize biotin [41]. On the other hand, the transcription of the genes involving fatty acid synthesis including *accBC, accD1, accD2, accD3, accD4*, and *fasA* [16, 42] showed no regular changes under the excessive biotin although biotin is the cofactor of acetyl-CoA carboxylase on the first committed step of fatty acid synthesis [16], indicating biotin excessive condition may have limited effects on varying gene expression involving fatty acid synthesis.

The fermentation and transcriptional analysis results suggest that the presence of excessive biotin in the CSH stimulates the fast transport of biotin into the cells, then strengthens the cell structure and decreases the flux to glutamate synthesis. Finally, the glutamate secretion and the accumulation of glutamic acid in corn stover hydrolysate are strongly suppressed.

### Biotin content of lignocellulose in biorefining chain

**Fig. 5:**
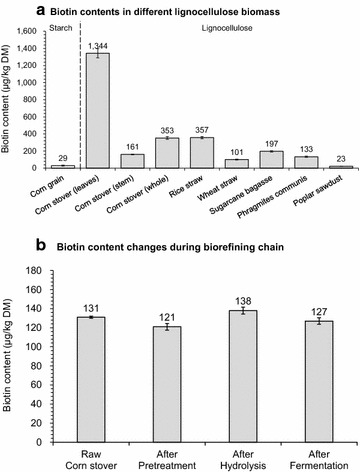
Biotin contents in different lignocellulose biomass and biotin content changes during biorefining steps. **a** Biotin contents in different lignocellulose biomass. **b** Biotin content changes during biorefining chain. The measured biotin concentration in the hydrolysate and fermentation broth was transformed to the amount of biotin content per kg of the pretreated corn stover. Mean values were presented with error bars representing at least two standard deviations

To investigate why so much biotin was retained in the corn stover hydrolysate. We recorded the biotin content of corn stover during the biorefining chain of pre-handling, pretreatment, detoxification, enzymatic hydrolysis, as well as glutamic acid fermentation (Fig. 5). The newly harvested corn stover contained the maximum biotin at 353 ± 16 μg per kg of dry corn stover matter (DM), more than one order of magnitude greater than the biotin in corn grain (29 ± 6 μg/kg DM) (Fig. 5a) and close to the level of the typical fermentation nutrients additives corn steep liquor (744 ± 15 μg/kg DM), peanut meal (1790 μg/kg DM), and yeast extract (1000 μg/kg DM) [26]. This rich existence of biotin was also observed in corn stover collected from other places, for example, 282 ± 11 μg/kg from Bayan Nur, Inner Mongolia, China, in fall 2015 and 330 ± 15 μg/kg from Tongliao, Inner Mongolia, China, in fall 2016. The pre-handling of virgin corn stover by water washing to remove the field dirt, sands and metal pieces led to the biotin loss of 60% to 131 ± 1 μg/kg DM, but the loss is certainly avoidable in the large industrial practice where mechanic vibration and cyclone of corn stover are applied, instead of water washing in small bench operation [43].

In the core steps of biorefining process of pretreatment, detoxification, hydrolysis, and fermentation, the biotin content maintained approximately constant (Fig. 5b). In the dry acid pretreatment step (175 °C for 5 min with addition of 2.5% sulfuric acid), approximately 92% (121 ± 4 μg/kg) of biotin was maintained largely due to no wastewater generation in this specific pretreatment method. The biotin content after biodetoxification and enzymatic hydrolysis was 138 ± 4 μg/kg DM, a slight increase to that of the pretreated corn stover, perhaps due to the reduced inhibition by biodetoxification on the cell growth of the biotin assay strain *L. plantarum*. No biotin was detected in the *A. resinae* ZN1 cells and the cellulase enzyme solution. In the fermentation step, the biotin in corn stover hydrolysate was completely transported into the *C. glutamicum* cells (Fig. 3), but the total amount of biotin maintained almost the same since no biotin metabolized by the cell.

Due to the specific characteristic of our biorefinery process and the high chemical stability of biotin under the treatment by sulfuric acid, a stable and well conserved mass balance of biotin in the biorefining chain emerged which caused a negative scenario for glutamic acid production but created a new sight for the biorefinery process.

### Extended identification of biotin and vitamin B compounds in general lignocellulose and biorefining chain

The biotin content measurement was extended to different lignocellulose biomass. The selected lignocellulose biomass included corn stover, rice straw, wheat straw, and sugarcane bagasse as the typical agricultural biomass, *Phragmites communis* reeds as the typical reeds biomass, and Italian poplar sawdust as the typical wood biomass. Biotin content in the starch biomass (corn) was also assayed as the control (Fig. 5a). The results show that except poplar sawdust with relative low biotin content (23 ± 0.2 μg/kg DM), corn stover, rice straw, wheat straw, sugarcane bagasse, and *P. communis* reeds contained as high as 353 ± 16, 357 ± 13, 101 ± 5, 197 ± 8, and 133 ± 7 μg/kg dry biomass matter (DM), respectively. Biotin in the corn leaves even reached 1344 ± 54 μg/kg DM, almost one order of magnitude greater than that in the stem of corn stover (161 ± 4 μg/kg DM) and two orders of magnitude greater than that in corn grain (29 ± 6 μg/kg). Perhaps due to the plastids in leaves requires more biotin as the cofactor for acetyl-CoA carboxylase in de novo fatty acid synthesis [44], those biomass samples contain more plastids tend to have a higher content of biotin, while the poplar sawdust contains the least amount of plastids thus contains relative low biotin content. This common phenomenon of rich biotin in the high plastids containing lignocellulose biomass which caused no glutamic acid accumulation by *C. glutamicum* may be one of the reasons why few studies concerning glutamic acid production use lignocellulose biomass.

Biotin has been found in various crop biomass, fruits and dairy products such as wheat, corn, potato, beet and cane molasses, peanut meal, grape, milk and so on [44, 45]. This study gave the first insight on the excessive content of biotin in lignocellulose biomass. In plants, biotin existed either in its free form in cytosol or the bound form as the cofactor of carboxylases in organelles [46]. The biotin-bound carboxylases are required for cellular metabolism such as amino acid catabolism, fatty acid synthesis, and carbohydrates gluconeogenesis [47], while the free biotin may act as a reserve pool for plant cells [48]. In biotin auxotrophic bacteria such as *C. glutamicum*, free biotin content generally is very low [26] and the uptake of biotin from extracellular sources is required for their cell growth and metabolism while glutamic acid accumulation failed when biotin is excessive as in corn stover hydrolysate.

Besides biotin, we expected that certain levels of vitamin B compounds may exist in lignocellulose biomass, besides biotin. The quantitative assay was extended on the complete vitamin B members in virgin corn stover and in their content profile of biorefinery processing steps (Table 1). The vitamin B contents reported in starch biomass (corn and rice) were taken as the controls of the measurement. All eight vitamin B compounds were detected in virgin corn stover and the contents were comparable to that in starch biomass. In the pre-handling step, the water-soluble thiamin, riboflavin, niacin, pantothenate, pyridoxine, folate, and cobalamin were washed out by 67, 48, 38, 22, 44, 8, and 14%, respectively. As mentioned above, water washing is only a bench approach for removal of field dirt and not practiced in industrial processes [43], thus the loss could be reasonably neglected. In the dry acid pretreatment step, thiamin (500 μg/kg DM) and riboflavin (4200 μg/kg DM) increased more than twofold, perhaps due to the release of their bound form by the harsh pretreatment action of sulfuric acid hydrolysis at high temperature (175 °C), comparing to the relatively weak extraction in the assay methods used. On the other hand, significant decrease of pyridoxine (500–200 μg/kg DM), niacin (4600–2600 μg/kg DM), and pantothenate (988–130 μg/kg DM) were observed in the pretreatment step, due to the unstable properties of the compounds. Folic acid in the virgin corn stover was high, but it was degraded significantly after pretreatment (below the detection line, 20 μg/kg DM). Cobalamin is synthesized solely in prokaryotic microorganisms [49], therefore the minor cobalamin content in corn stover should come from the contamination from bacteria during the plant growth period.

**Table 1:** Vitamin B contents in the raw corn stover during biorefining chain (μg/kg DM)

Vitamin B members	In virgin corn stover	After washing	After pretreatment	In starch biomass^a^
Thiamin (vitamin B1)	600	200	500	4100 (Rice) [30]
Riboflavin (vitamin B2)	2100	1100	4200	2600 (Corn) [30]
Niacin (vitamin B3)	7400	4600	2600	16000 (Corn) [49]
Pantothenate (vitamin B5)	1270	988	130	2000 (Rice) [30]
Pyridoxine (vitamin B6)	900	500	200	20000 (Corn) [50]
Biotin (vitamin B7)	353 ± 16	131 ± 1	121 ± 4	< 0.15 (Corn) [45]
Folic acid (vitamin B9)	118	108	< 20^b^	19 (Rice) [51]
Cobalamin (vitamin B12)	7	6	< 1^b^	N/A [30]

The standard deviations of biotin were derived from at least two independent determinations

^a^ Vitamin B compounds contents in starch biomass were referred to the references in the list

^b^ The contents were below the detection line

Vitamin B compounds are the essential additives in various fermentations such as for the production of ethanol [24], lactic acid [25], and amino acids [22, 23]. Compared to cereal biomass (rice and corn) [45, 49–52], thiamin, riboflavin, niacin, pantothenate, pyridoxine, and biotin in the pretreated corn stover still retained at certain level and may act as fermentation nutrients for elevating the fermentability of biorefining strains. Few examples include the enhancement of biotin on the anti-oxidative activity of *Pichia guilliermondii* to improve cellulosic ethanol fermentation [24], and the cell growth enhancement of *Pediococcus acidilactici* in corn stover hydrolysate than that in MRS medium in the cellulosic lactic acid fermentation [11]. In addition, our glutamic acid fermentation with enhanced cell growth in corn stover hydrolysate did not need to add additional nutrients except nitrogen resources. These results demonstrated that the pretreatment of lignocellulose not only generates the inhibitor compounds, but also liberates the nutrients such as vitamin B compounds in lignocellulose biomass and facilitates the biorefining fermentation for production of biofuels and bio-based products.

This study provides a new insight on supplementation of vitamin B nutrients or more plant-derived components into biorefining fermentations for enhancement of fermentability or yield in biorefining chains. Based on this perspective, biorefining chain of lignocellulose should carefully tune the process intensity for preservation of biotin and other vitamin B compounds, besides the conventional consideration of extensively disrupting the supermolecular structure of lignocellulose, generating less toxic inhibitor compounds, and other practical issues on reducing wastewater generation and energy consumption.

## Conclusions

This study provides the first insight into the discovery of surprisingly high content of biotin and other vitamin B compounds in various lignocellulose feedstocks during the biorefining process. The excessive biotin is highly stable in lignocellulose biorefining chain and plays the determinant role on the cellulosic glutamic acid fermentation. High titer cellulosic glutamic acid was produced from corn stover feedstock by penicillin induction on *Corynebacterium glutamicum* with the potential to compete with corn-derived glutamic acid production. This study also reveals that lignocellulose biorefining creates not only inhibitors, but also nutrients for fermentations.

## Methods

### Raw materials

Corn stover and wheat straw were harvested from Dancheng, Henan, China, in fall 2013. Rice straw was harvested from Chuzhou, Anhui, China, in summer 2014. Sugarcane bagasse was obtained from a sugar plant of Beihai, Guangxi, China, in summer 2014. *Phragmites communis* reeds and poplar sawdust were harvested from Yuncheng, Shanxi, China, in summer 2014. The collected lignocellulose biomass was water washed and sedimented to remove field dirt, sands, and metal pieces, then air dried and milled using a beater pulverizer to pass through 10-mm apertures in diameter. After these treatments, the raw biomass contained about 10% moisture with no other volatile solids. The composition of the raw biomass was determined using the two-step sulfuric acid hydrolysis method according to the National Renewable Energy Laboratory (NREL) protocols [53, 54] and summarized in Additional file 1: Table S2.

### Strains and culture media

*Corynebacterium glutamicum* S9114 was purchased from Shanghai Industrial Institute of Microorganism (SIIM, http://www.gsy-siim.com/), Shanghai, China, with the storage code of SIIM B460. This strain also stored at China Center of Industrial Culture Collection (CICC, http://www.china-cicc.org/), Beijing, China, with the storage number of CICC 20935. The culture media include: (1) LB agar containing 1% of peptone, 0.5% of yeast extract, 0.5% of NaCl, and 1.7% of agar at pH 7.0; (2) preculture medium containing 25 g/L of glucose, 1.5 g/L of KH_2_PO_4_, 0.6 g/L of MgSO_4_, 2.5 g/L of urea, 2.0 mg/L of FeSO_4_, 2.0 mg/L of MnSO_4_, 25 g/L of corn steep liquor (CSL); (3) seed culture medium containing 25 g/L of glucose, 1.5 g/L of KH_2_PO_4_, 0.6 g/L of MgSO_4_, 2.5 g/L of urea, 2.0 mg/L of FeSO_4_, 2.0 mg/L of MnSO_4_, 5 g/L of CSL; (4) Biotin-limited complex medium, containing 60 g/L of glucose, 1 g/L of KH_2_PO_4_, 0.6 g/L of MgSO_4_, 3 g/L of urea, 2.0 mg/L of FeSO_4_, 2.0 mg/L of MnSO_4_, 0.5 g/L of CSL; (5) Biotin rich complex medium, containing 60 g/L of glucose, 1 g/L of KH_2_PO_4_, 0.6 g/L of MgSO_4_, 3 g/L of urea, 2.0 mg/L of FeSO_4_, 2.0 mg/L of MnSO_4_, 5.0 g/L of CSL. All the media were autoclaved in 115 °C for 20 min before use.

The biodetoxification fungus *Amorphotheca resinae* ZN1 was isolated in our previous study [55] and stored in China General Microorganism Collection Center (CGMCC, http://www.cgmcc.net/), Beijing, China, with the register code of 7452. *A. resinae* ZN1 was cultured on potato dextrose agar (PDA) medium containing 200 g of potato juice, 20 g of glucose and 17 g of agar in 1 L of deionized water.

### Enzymes and reagents

Cellulase enzyme Youtell #6 was purchased from Hunan Youtell Biochemical Co., Yueyang, Hunan, China. The protein concentration was 90 mg/g dry cellulase solid matter (DM). The filter paper activity was 135 FPU/g DM using the NREL protocol LAP-006 [56]. The cellobiase activity was 344 CBU/g DM using the method mentioned previously [57]. Avidin was purchased from Aladdin Industrial Co., Shanghai, China, with the labeled purity of 10–15 units per mg protein, and diluted into 1 mg/L solution and sterile filtrated before use. Biotin was purchased from Aladdin Industrial Co., Shanghai, China, with the labeled purity of more than 98%, and diluted into 1 mg/L solution and sterile filtrated before use. Penicillin was purchased from New Probe Biochem Co., Beijing, China, with the labeled titer of 1650 U/mg, and diluted into the 50 mg/mL penicillin solution for induction experiment. Corn steep liquor (CSL) was purchased from Xiwang Group Co., Zouping, Shandong, China. Other general chemicals used in this study were analytical grade and purchased from local supplier Lingfeng Chemical Reagent Co., Shanghai, China.

### Pretreatment and biodetoxification operations

Corn stover was pretreated using dry acid pretreatment method [58, 59]. Briefly, 1200 g of feedstock (dry base) and approximately 600 g of 5% (w/w) dilute sulfuric acid solution (depending on the moisture content of the feedstock) were co-currently fed into the pretreatment reactor for 3 min at the solids/liquid ratio of 2:1 (w/w). The reactor was 20 L in the inner volume and thermally insulated. A single helical ribbon impeller was installed under the mild agitation rate (50 rpm). The sulfuric acid concentration in the dilute acid solution was adjusted in a narrow range according to the measured moisture content of the feedstocks. The saturated water steam (1.6 MPa) was produced from a steam generator machine (HX-36D, Huazheng Boiler Co., Shanghai, China). The pretreatment operation started when the hot steam was jetted onto the feedstock bulk in the reactor to 175 ± 1 °C for 5 min under the mild helical agitation (50 rpm). Then the pretreated solid feedstocks were discharged gravitationally from the bottom outlet port. All the dilute acid solution and the condensed water were completely adsorbed into the solids to form approximately 50% (w/w) of the dry pretreated feedstock solids with the pH around 2.0, and no free wastewater stream was generated. The pretreated corn stover contained 29.7 mg of glucose, 155.1 mg of xylose, 5.3 mg of furfural, 3.5 mg of 5-hydroxymethylfurfural (HMF), 24.8 mg of acetic acid, 0.06 mg of 4-hydroxybenzaldehyde (HBA), 3.3 mg of vanillin, and 2.2 mg of syringaldehyde per gram of the dry pretreated corn stover matter (DM). Furfural, HMF and acetic acid are the volatile compounds in the pretreated corn stover.

The pretreated corn stover was biodetoxified to remove the inhibitors as described before [13, 55]. Briefly, the residual sulfuric acid in the pretreated biomass solids was neutralized to 5.5 by the addition of 20% (w/w) Ca(OH)_2_ suspension slurry. The pretreated biomass solids were briefly milled by a disk milling machine (PSB-80JX, Fleck Co., Nantong, Jiangsu, China) to remove the extra-long fibers to avoid the blockage of pipelines and valves in the downstream flow of the hydrolysate slurry and broth. *A. resinae* ZN1 spores were collected from PDA slant, then inoculated onto the freshly pretreated corn stover solids and cultured for 5 days at 28 °C as the seeds for biodetoxification. The *A. resinae* ZN1 seed solids were inoculated at 10% (w/w) onto the freshly pretreated corn stover and incubated at 28 °C under the aeration of 1.0 vvm (based on the pretreated corn stover volume) for 48 h and then sterilized at 115 °C for 20 min before use. No fresh water or extra nutrients were added during biodetoxification. Furfural and HMF were completely removed by biodetoxification and the residual inhibitors in the detoxified corn stover was reduced to 21.9 mg of acetic acid, 0.02 mg of HBA, 1.1 mg of vanillin, and 0.4 mg of syringaldehyde per gram of the dry detoxified corn stover matter (DM).

### Corn stover hydrolysate preparation

The fermentation corn stover hydrolysate (CSH) was prepared by hydrolyzing the dry acid pretreated and biodetoxified corn stover at different solids content (15%, 20%, 25%, w/w) under the conditions of 10 mg cellulase proteins per gram of dry corn stover matter (mg/g DM), 50 °C, pH 4.8 for 48 h. The hydrolysate slurries were centrifuged at 16,125×*g* for 10 min to remove the solid residues and obtain the clear supernatant hydrolysate. The hydrolysate was autoclaved in 115 °C for 20 min and then sterile filtrated before use. The non-detoxified CSH used for inhibitor tolerance assay was prepared from the pretreated corn stover under 15% (w/w) solids content without detoxification treatment. The compositions were summarized in Additional file 1: Table S3.

Biotin binding protein, avidin, was used to prepared the biotin free CSH. Considering the miss binding of biotin by avidin would happened in the complex CSH, different dosage of avidin solution (1 mg/L, sterile filtrated) was first added to the fermentation CSH (15% solids content, w/w) followed by the incubation at 30 °C and 200 rpm for 2 h before use to test the cell growth and the glutamic acid accumulation. Finally, the optimal dosage equivalent to 20 mg/L avidin (equivalent to 200 units/L of CSH) was chosen to prepare the biotin free CSH. The suboptimal biotin re-supplemented CSH was prepared by adding 30 μL of 1 mg/L sterile filtrated biotin solution (equivalent to 1.0 μg/L CSH of pure biotin) to the biotin free CSH. The excessive biotin re-supplemented CSH was prepared by adding 690 μL of 1 mg/L sterile filtrated biotin solution (equivalent to 23.0 μg/L CSH of pure biotin) to the biotin free CSH.

### Glutamic acid fermentation

*Corynebacterium glutamicum* S9114 was evolutionarily adapted in the non-detoxified CSH by continuously transfer every 24 h or 147 times at 10% (v/v) inoculum ratio, 30 °C, pH 7.0, 200 rpm until the cell growth and glucose consumption were stable. Cells grew on LB petri dish at 30 °C for 24–36 h, then a single colony was picked up, inoculated into the 250 mL-Erlenmeyer flasks containing 30 mL preculture medium flask. The cells were cultured in the preculture medium for 10 h at 30 °C and 200 rpm. Then 1.5 mL of the broth was transferred into 30 mL of the seed medium at 30 °C, 200 rpm for 8 h and consequently transferred into the fermentation medium. pH was maintained at 7.0 by adding 20% of the sterilized urea solution. Samples were withdrawn at regular intervals for analysis.

The batch fermentation in fermentor was carried out in a 3 L fermentor (Biotech-3BG-4, Baoxing Co., China) at 32 °C containing 800 mL of corn stover hydrolysate with addition of 5 g/L of (NH_4_)_2_SO_4_ into the initial hydrolysate. The inoculum ratio of the *C. glutamicum* seed culture was 10% (v/v), the pH was 7.0 by addition of 20% (w/v) ammonium hydroxide solution, and the aeration rate was 1.4 vvm. Dissolved oxygen (DO) was regulated by changing the agitation rate to 10% of the saturation in the exponential phase of cell growth and 40% of saturation in the glutamic acid accumulation stage. 2 mL of penicillin solution (50 mg/L) was added when the optical density at 600 nm (OD_600_) was in the range of 8–9 for induction of glutamic acid secretion. All fermentations were carried out in duplicate. The standard derivation was indicated by error bars.

### Analytical methods

Glucose, glutamic acid and lactic acid were analyzed using the SBA-40D biosensor (Biology Institute, Shandong Academy of Sciences, Jinan, Shandong, China). Acetic acid, xylose, furfural, and HMF were analyzed on HPLC (LC-20AD, refractive index detector RID-10A, Shimadzu, Kyoto, Japan) with a Bio-Rad Aminex HPX-87H column (Bio-rad, Hercules, CA, USA) at 65 °C and 5 mM H_2_SO_4_ solution as the mobile phase at the flow rate of 0.6 mL/min. Phenolic compounds were analyzed on HPLC (UV/Vis detector SPD-20A, at 270 nm, Shimadzu, Kyoto, Japan) with a YMC-Pack ODS-A column (YMC Co., Kyoto, Japan) at 35 °C as mentioned before [13].

Cell growth was indicated by optical density at 600 nm (OD_600_) by the spectrophotometer Beckman Coulter DU800 (Beckman, Brea, CA, USA). The dry cell weight (DCW) was transformed 1 unit of OD_600_ to approximately 0.4 mg/mL of the dry cell weight.

### RT-qPCR assay

Transcription of *C. glutamicum* was quantitated using real-time quantitative PCR (RT-qPCR) in the three media: the biotin-limited complex medium (containing 0.5 g/L CSL) used as the basic control, the biotin rich complex medium (containing 5.0 g/L CSL), and the CSH (15% solids content, w/w). *C. glutamicum* was cultured in flask and the cells were harvested at 10 h, then immediately frozen in liquid nitrogen and the total RNA was extracted using Trizol reagent kit (Invitrogen, Carlsbad, CA, USA). Purity and concentrations of the RNA samples were indicated by the ratio of OD_260/280_ readings using NanoDrop ND-1000 spectrophotometer (NanoDrop Technologies Inc., Wilmington, DE, USA). Primers of the selected genes are shown in Additional file 1: Table S4 based on the genome sequence of *C. glutamicum* S9114 in National Center of Biotechnology Information (NCBI) GenBank database (https://www.ncbi.nlm.nih.gov/nuccore/NZ_AFYA00000000.1). The first strand of cDNA was synthesized using cDNA synthesis kit (Toyobo Co., Osaka, Japan). The qPCR reaction was run on a CFX96 Real-Time System with C10000 Thermal Cycler (Bio-Rad, Hercules, CA, USA). The RT-qPCR was carried out at 95 °C for 1 min, then 40 cycles at 95 °C for 15 s, 55.4 °C for 15 s, and 72 °C for 30 s using the SYBR Green Real-time PCR Master Mix. The 16 s ribosomal RNA (CGS9114_RS11955) was used as the internal control for data acquisition and normalization. The relative gene expression data in the biotin rich complex medium and the CSH (15% solid loading, w/w) were normalized by the gene expression data in the biotin-limited medium using the 2^−ΔΔCt^ method [60]. The fold change ≥ 2.0 or ≤ 0.5 was set to be the criteria for the differential expression genes.

### Vitamin B compound assay

Biotin was measured using VitaFast Kit (R-Biopharm AG, Darmstadt, Germany) by measuring the cell growth of cell mass of a biotin auxotrophic strain *Lactobacillus plantarum* ATCC 8014 under varied biotin content [61] which was in accordance with international norms. The extraction and determination method was according to the method provided by the kit. For the intracellular biotin determination, cells were harvested, centrifuged at 13,000×*g* for 5 min, washed twice by 0.85% NaCl solution. The washed cells were re-suspended in 1 M sulfuric acid solution and autoclave at 121 °C for 30 min to liberate bound biotin and then the pH was adjusted to 4.5 before determination.

Other vitamin B compounds of the samples were determined in the Shanghai Technical Center for Animal Plant and Food Inspection and Quarantine (http://spzx.shciq.gov.cn/), Shanghai, China, which based on the standard methods with the values of the Coefficient of Variances (CVs) less than 10%. Thiamin (vitamin B1) was assayed based on fluorescence analysis method as described previously [62]. Riboflavin (vitamin B2) and folic acid (vitamin B9) were assayed based on the cell growth of the riboflavin and folic acid dependent strain *Lactobacillus casei* ATCC 7469 [28, 63]. Niacin (vitamin B3) and pantothenate (vitamin B5) were assayed based on the cell growth of a niacin and pantothenate auxotrophic strain *Lactobacillus plantarum* ATCC 8014 [63, 64]. Pyridoxine (vitamin B6) and cobalamin (vitamin B12) was assayed based on the cell growth of the pyridoxine dependent strain *Saccharomyces carlsbergensis* ATCC 9080 [30] and the cobalamin dependent strain *Lactobacillus leichmannii* ATCC 7830 [63, 65].

## Additional file

**Additional file 1: Table S1.** Expressions of genes involving glutamic acid accumulation of *C. glutamicum* S9114 in biotin rich conditions. **Table S2.** Compositions of different lignocellulose biomass. **Table S3.** Concentrations of glucose, xylose and inhibitor compounds in different corn stover hydrolysate (CSH). **Table S4.** Primers used in real-time quantitative PCR (RT-qPCR) assay.

## Abbreviations

CSH: corn stover hydrolysate
CSL: corn steep liquor
HMF: 5-hydroxymethylfurfural
HBA: 4-hydroxybenzaldehyde
DCW: dry cell weight
RT-qPCR: real-time quantitative PCR
ODHC: α-ketoglutarate dehydrogenase complex
MscCG: mechanosensitive channel
DM: dry matter
NREL: National Renewable Energy Laboratory
SIIM: Shanghai Industrial Institute of Microorganism
CICC: China Center of Industrial Culture Collection
CGMCC: China General Microorganism Collection Center
LAP: laboratory analytical procedure
PDA: potato dextrose agar
FPU: filter paper unit
CBU: cellobiase unit
DO: dissolved oxygen
OD_600_: optical density at wavelength (λ) 600 nm
HPLC: high-performance liquid chromatography
cDNA: complementary DNA
CVs: coefficient of variances

## References

[1] Woo HM, Park JB (2014). Recent progress in development of synthetic biology platforms and metabolic engineering of *Corynebacterium glutamicum*. J Biotechnol.

[2] Werpy T, Petersen G (2004). Top value added chemicals from biomass-vol. I: results of screening for potential candidates from sugars and synthesis gas.

[3] Jonsson LJ, Alriksson B, Nilvebrant NO (2013). Bioconversion of lignocellulose: inhibitors and detoxification. Biotechnol Biofuels.

[4] Zhang H, Zhang J, Bao J (2016). High titer gluconic acid fermentation by *Aspergillus niger* from dry dilute acid pretreated corn stover without detoxification. Bioresour Technol.

[5] Becker J, Wittmann C (2012). Bio-based production of chemicals, materials and fuels —*Corynebacterium glutamicum* as versatile cell factory. Curr Opin Biotech.

[6] Jojima T, Inui M, Yukawa H, Yukawa H, Inui M (2013). Biorefinery applications of *Corynebacterium glutamicum*. *Corynebacterium glutamicum*: biology and biotechnology.

[7] Gopinath V, Meiswinkel TM, Wendisch VF, Nampoothiri KM (2011). Amino acid production from rice straw and wheat bran hydrolysates by recombinant pentose-utilizing *Corynebacterium glutamicum*. Appl Microbiol Biotechnol.

[8] Meiswinkel TM, Gopinath V, Lindner SN, Nampoothiri KM, Wendisch VF (2013). Accelerated pentose utilization by *Corynebacterium glutamicum* for accelerated production of lysine, glutamate, ornithine and putrescine. Microb Biotechnol.

[9] Liu G, Zhang Q, Li H, Qureshi AS, Zhang J, Bao X, Bao J (2018). Dry biorefining maximizes the potentials of simultaneous saccharification and co-fermentation for cellulosic ethanol production. Biotechnol Bioeng.

[10] Wang J, Gao Q, Zhang H, Bao J (2016). Inhibitor degradation and lipid accumulation potentials of oleaginous yeast *Trichosporon cutaneum* using lignocellulose feedstock. Bioresour Technol.

[11] Zhao K, Qiao Q, Chu D, Gu H, Dao TH, Zhang J, Bao J (2013). Simultaneous saccharification and high titer lactic acid fermentation of corn stover using a newly isolated lactic acid bacterium *Pediococcus acidilactici* DQ2. Bioresour Technol.

[12] Zhou PP, Meng J, Bao J (2017). Fermentative production of high titer citric acid from corn stover feedstock after dry dilute acid pretreatment and biodetoxification. Bioresour Technol.

[13] He Y, Zhang J, Bao J (2016). Acceleration of biodetoxification on dilute acid pretreated lignocellulose feedstock by aeration and the consequent ethanol fermentation evaluation. Biotechnol Biofuels.

[14] Shiio I, Otsuka SI, Takahashi M (1962). Effect of biotin on the bacterial formation of glutamic acid. I. Glutamate formation and cellular permeability of amino acids. J Biol Chem.

[15] Takinami K, Yamada Y, Okada H (1966). Biochemical effects of fatty acid and its derivatives on l-Glutamic acid fermentation: Part IV biotin content of growing cells of *Brevibacterium lactofermentum*. Agric Biol Chem.

[16] Gande R, Dover LG, Krumbach K, Besra GS, Sahm H, Oikawa T, Eggeling L (2007). The two carboxylases of *Corynebacterium glutamicum* essential for fatty acid and mycolic acid synthesis. J Bacteriol.

[17] Eggeling L, Krumbach K, Sahm H (2001). l-glutamate efflux with *Corynebacterium glutamicum*: why is penicillin treatment or tween addition doing the same?. J Microbiol Biotechnol.

[18] Takinami K, Yoshii H, Tsuri H, Okada H (1965). Biochemical effects of fatty acid and its derivatives on l-Glutamic acid fermentation: Part III Biotin-tween 60 relationship in the accumulation of l-glutamic acid and the growth of *Brevibacterium lactofermentum*. Agric Biol Chem.

[19] Nara T, Samejima H, Kinoshita S (1964). Effect of penicillin on amino acid fermentation. Agric Biol Chem.

[20] Radmacher E, Stansen KC, Besra GS, Alderwick LJ, Maughan WN, Hollweg G, Sahm H, Wendisch VF, Eggeling L (2005). Ethambutol, a cell wall inhibitor of *Mycobacterium tuberculosis*, elicits l-glutamate efflux of *Corynebacterium glutamicum*. Microbiology.

[21] Nakamura J, Hirano S, Ito H, Wachi M (2007). Mutations of the *Corynebacterium glutamicum* NCgl1221 gene, encoding a mechanosensitive channel homolog, induce l-glutamic acid production. Appl Environ Microbiol.

[22] Ko YT, Chipley JR (1984). Role of biotin in the production of lysine by *Brevibacterium lactofermentum*. Microbios.

[23] Park SH, Kim HU, Kim TY, Park JS, Kim SS, Lee SY (2014). Metabolic engineering of *Corynebacterium glutamicum* for l-arginine production. Nat Commun.

[24] Qi K, Xia XX, Zhong JJ (2015). Enhanced anti-oxidative activity and lignocellulosic ethanol production by biotin addition to medium in *Pichia guilliermondii* fermentation. Bioresour Technol.

[25] Nancib A, Nancib N, Meziane-Cherif D, Boubendir A, Fick M, Boudrant J (2005). Joint effect of nitrogen sources and B vitamin supplementation of date juice on lactic acid production by *Lactobacillus casei* subsp. *rhamnosus*. Bioresour Technol.

[26] Oura E, Suomalainen H (1982). Biotin-active compounds, their existence in nature and the biotin requirements of yeasts. J Inst Brew.

[27] Arcot J, Shrestha A (2005). Folate: methods of analysis. Trends Food Sci Tech.

[28] Goyer A (2010). Thiamine in plants: aspects of its metabolism and functions. Phytochemistry.

[29] Massey V (2000). The chemical and biological versatility of riboflavin. Biochem Soc Trans.

[30] Ollilainen V, Finglas PM, van den Berg H, de Froidmont-Gortz I (2001). Certification of B-group vitamins (B1, B2, B6, and B12) in four food reference materials. J Agric Food Chem.

[31] Gerstmeir R, Wendisch VF, Schnicke S, Ruan H, Farwick M, Reinscheid D, Eikmanns BJ (2003). Acetate metabolism and its regulation in *Corynebacterium glutamicum*. J Biotechnol.

[32] Li HW, Su QH, Li ZJ, Dai LY (2005). Comprehensive report on Chinese glutamic acid production trade. Acad Period Farm Prod Process.

[33] Abendroth C, Simeonov C, Pereto J, Antunez O, Gavidia R, Luschnig O, Porcar M (2017). From grass to gas: microbiome dynamics of grass biomass acidification under mesophilic and thermophilic temperatures. Biotechnol Biofuels.

[34] Waller JR, Anderson JK, Ulmer DC (1984). Use of avidin to prepare biotin-free culture media. Anal Biochem.

[35] Nampoothiri K, Hoischen C, Bathe B, Möckel B, Pfefferle W, Krumbach K, Sahm H, Eggeling L (2002). Expression of genes of lipid synthesis and altered lipid composition modulates l-glutamate efflux of *Corynebacterium glutamicum*. Appl Microbiol Biotechnol.

[36] Wijayarathna CD, Wachi M, Nagai K (2001). Isolation of *ftsI* and *murE* genes involved in peptidoglycan synthesis from *Corynebacterium glutamicum*. Appl Microbiol Biotechnol.

[37] Matsuhashi M, Wachi M, Ishino F (1990). Machinery for cell growth and division: penicillin-binding proteins and other proteins. Res Microbiol.

[38] Asakura Y, Kimura E, Usuda Y, Kawahara Y, Matsui K, Osumi T, Nakamatsu T (2007). Altered metabolic flux due to deletion of *odhA* causes L-glutamate overproduction in *Corynebacterium glutamicum*. Appl Environ Microbiol.

[39] Shimizu H, Tanaka H, Nakato A, Nagahisa K, Kimura E, Shioya S (2003). Effects of the changes in enzyme activities on metabolic flux redistribution around the 2-oxoglutarate branch in glutamate production by *Corynebacterium glutamicum*. Bioprocess Biosyst Eng.

[40] Schneider J, Peters-Wendisch P, Stansen KC, Gotker S, Maximow S, Kramer R, Wendisch VF (2012). Characterization of the biotin uptake system encoded by the biotin-inducible *bioYMN* operon of *Corynebacterium glutamicum*. BMC Microbiol.

[41] Peters-Wendisch P, Götker S, Heider SA, Komati RG, Nguyen AQ, Stansen KC, Wendisch VF (2014). Engineering biotin prototrophic *Corynebacterium glutamicum* strains for amino acid, diamine and carotenoid production. J Biotechnol.

[42] Radmacher E, Alderwick LJ, Besra GS, Brown AK, Gibson KJ, Sahm H, Eggeling L (2005). Two functional FAS-I type fatty acid synthases in *Corynebacterium glutamicum*. Microbiology.

[43] Humbird D, Davis R, Tao L, Kinchin C, Hsu D, Aden A, Schoen P, Lukas J, Olthof B, Worley M. Process design and economics for biochemical conversion of lignocellulosic biomass to ethanol: dilute-acid pretreatment and enzymatic hydrolysis of corn stover. NREL technical report NREL/TP-5100-47764, National Renewable Energy Laboratory, Golden, CO. 2011.

[44] Rawsthorne S (2002). Carbon flux and fatty acid synthesis in plants. Prog Lipid Res.

[45] Scheiner J, De Ritter E (1975). Biotin content of feedstuffs. J Agric Food Chem.

[46] Baldet P, Alban C, Axiotis S, Douce R (1992). Characterization of biotin and 3-methylcrotonyl-coenzyme a carboxylase in higher plant mitochondria. Plant Physiol.

[47] Alban C, Job D, Douce R (2000). Biotin metabolism in plants. Annu Rev Plant Phys.

[48] Baldet P, Alban C, Axiotis S, Douce R (1993). Localization of free and bound biotin in cells from green pea leaves. Arch Biochem Biophys.

[49] Zempleni J, Suttie JW, Gregory JF, Stover PJ (2013). Handbook of Vitamins.

[50] Carpenter KJ, Schelstraete M, Vilicich VC, Wall JS (1988). Immature corn as a source of niacin for rats. J Ethnopharmacol.

[51] Gregory J, Litherland SA (1986). Efficacy of the rat bioassay for the determination of biologically available vitamin B-6. J Nutr.

[52] Pfeiffer CM, Rogers LM, Bailey LB, Gregory JF (1997). Absorption of folate from fortified cereal-grain products and of supplemental folate consumed with or without food determined by using a dual-label stable-isotope protocol. Am J Clin Nutr.

[53] Sluiter A, Hames B, Ruiz R, Scarlata C, Sluiter J, Templeton D (2008). Determination of sugars, byproducts, and degradation products in liquid fraction process samples.

[54] Sluiter A, Hames B, Ruiz R, Scarlata C, Sluiter J, Templeton D, Crocker D (2012). Determination of structural carbohydrates and lignin in biomass.

[55] Zhang J, Zhu Z, Wang X, Wang N, Wang W, Bao J (2010). Biodetoxification of toxins generated from lignocellulose pretreatment using a newly isolated fungus, *Amorphotheca resinae* ZN1, and the consequent ethanol fermentation. Biotechnol Biofuels.

[56] Adney B, Baker J (1996). Measurement of cellulase activities.

[57] Ghose TK (1987). Measurement of cellulase activities. Pure Appl Chem.

[58] Zhang J, Wang X, Chu D, He Y, Bao J (2011). Dry pretreatment of lignocellulose with extremely low steam and water usage for bioethanol production. Bioresour Technol.

[59] He Y, Zhang J, Bao J (2014). Dry dilute acid pretreatment by co-currently feeding of corn stover feedstock and dilute acid solution without impregnation. Bioresour Technol.

[60] Livak KJ, Schmittgen TD (2001). Analysis of relative gene expression data using real-time quantitative PCR and the 2^−ΔΔCt^ method. Methods.

[61] Zhang H, Lan F, Shi Y, Wan ZG, Yue ZF, Fan F, Lin YK, Tang MJ, Lv JZ, Xiao T, Yi C (2014). A “three-in-one” sample preparation method for simultaneous determination of B-group water-soluble vitamins in infant formula using VitaFast^®^ kits. Food Chem.

[62] MacBride DE, Wyatt C (1983). Evaluation of a modified AOAC determination for thiamin and riboflavin in foods. J Food Sci.

[63] Bell JG (1974). Microbiological assay of vitamins of the B groups in foodstuffs. Lab Pract.

[64] Walsh JH (1980). A comparison of microbiological and radioimmunoassay methods for the determination of pantothenic acid in foods. J Food Biochem.

[65] Shaw W, Bailey G (1974). Evaluation of two vitamin B12 assay kits and *L. leichmannii* bioassay. Clin Biochem.

