# Supplementary material for: Rich biotin content in lignocellulose biomass plays the key role in determining cellulosic glutamic acid accumulation by Corynebacterium glutamicum
Source: Biotechnol Biofuels. 2018 May 10;11:132. doi: 10.1186/s13068-018-1132-x (PMC5944095; doi:10.1186/s13068-018-1132-x)
Supplement: Supplementary file 1 — Additional file 1: Table S1. Expressions of genes involving glutamic acid accumulation of C. glutamicum S9114 in biotin rich conditions. Table S2. Compositions of different lignocellulose biomass. Table S3. Concentrations of glucose, xylose and inhibitor compounds in different corn stover hydrolysate (CSH). Table S4. Primers used in real-time quantitative PCR (RT-qPCR) assay. [file 13068_2018_1132_MOESM1_ESM.docx]

**Tables**

**Table S1.** Expressions of genes involving glutamic acid accumulation of *C. glutamicum* S9114 in biotin rich conditions.

| **Proteins** | **Genes** | **Locus_tag** | **Relative gene expression level** | |
| --- | --- | --- | --- | --- |
|  |  |  | **In biotin rich complex medium** | **In the CSH (15% solids content, w/w)** |
| **Phospholipid synthesis** |  |  |  |  |
| Cyclopropane-fatty-acyl-phospholipid  synthase | *ufaA* | CGS9114_RS06985 | 2.44 ± 0.77 | 4.32 ± 0.84 |
| Phosphatidylglycerophosphate synthase | *pgsA1* | CGS9114_RS06020 | 9.00 ± 3.00 | 17.65 ± 4.09 |
| Phosphatidylinositol α-mannosyltransferase | *pima* | CGS9114_RS07945 | 1.24 ± 0.31 | 5.48 ± 1.47 |
| **Peptidoglycan synthesis** |  |  |  |  |
| Cell division protein FtsI | *ftsI* | CGS9114_RS11420 | 3.60 ± 0.54 | 2.71 ± 0.27 |
| Cell division protein FtsI | *ftsI* | CGS9114_RS07835 | 2.16 ± 0.06 | 7.16 ± 0.09 |
| D-alanyl-D-alanine carboxypeptidase | *dacB* | CGS9114_RS00725 | 0.94 ± 0.02 | 2.45 ± 0.48 |
| D-alanyl-D-alanine carboxypeptidase | *dac* | CGS9114_RS13375 | 1.43 ± 0.39 | 0.87 ± 0.31 |
| **Glutamate synthesis** |  |  |  |  |
| α-ketoglutarate decarboxylase | *odhA* | CGS9114_RS03450 | 3.44 ± 0.32 | 5.20 ± 1.64 |
| Dihydrolipoamide acetyltransferase | *sucB* | CGS9114_RS08045 | 2.47 ± 0.42 | 2.60 ± 0.18 |
| Pyridine nucleotide-disulfide  oxidoreductase | *lpdA* | CGS9114_RS13335 | 0.80 ± 0.13 | 0.75 ± 0.18 |
| Dihydrolipoyl dehydrogenase | *lpd* | CGS9114_RS04910 | 1.51 ± 0.21 | 1.67 ± 0.28 |
| Glutamate dehydrogenase | *gdh* | CGS9114_RS12025 | 0.70 ± 0.13 | 0.87 ± 0.09 |
| Glutamate dehydrogenase (NADP^+^  dependent) | *gdh* | CGS9114_RS07420 | 1.47 ± 0.36 | 0.73 ± 0.06 |
| **Glutamate secretion** |  |  |  |  |
| Mechanosensitive channel MscCG^a^ | *yggB* | CGS9114_RS01440 | 0.32 ± 0.16 | 0.17 ± 0.04 |
| **Biotin transporter** |  |  |  |  |
| Biotin biosynthesis protein BioY | *bioY* | CGS9114_RS11175 | 7.16 ± 0.94 | 5.09 ± 0.69 |
| Cobalt ABC transporter ATP-binding  protein | *bioM* | CGS9114_RS11180 | 16.6 ± 1.78 | 13.19 ± 1.00 |
| ABC transporter permease | *bioN* | CGS9114_RS11185 | 5.14 ± 0.90 | 3.11 ± 0.29 |
| **Fatty acid synthesis** |  |  |  |  |
| Acetyl-/propionyl-CoA carboxylase subunit  α | *accBC* | CGS9114_RS13275 | 2.47 ± 0.64 | 2.07 ± 0.43 |
| Methylmalonyl-CoA carboxyltransferase^b^ | *accD1* | CGS9114_RS13235 | 1.13 ± 0.36 | 0.08 ± 0.02 |
| Methylmalonyl-CoA carboxyltransferase^c^ | *accD2* | CGS9114_RS13240 | 2.01 ± 0.19 | 1.53 ± 0.13 |
| Acetyl-CoA carboxylase subunit β | *accD3* | CGS9114_RS13505 | 1.11 ± 0.57 | 0.88 ± 0.17 |
| Acetyl-CoA carboxylase subunit β | *accD4* | CGS9114_RS12340 | 2.44 ± 0.24 | 5.38 ± 0.58 |
| Fatty acid synthase | *fasA* | CGS9114_RS13530 | 1.37 ± 0.33 | 0.54 ± 0.04 |

Standard deviations were derived from at least three independent determinations

^a^ 99% gene similarity to *NCgl1221* gene encoding mechanosensitive channel MscCG in *C. glutamicum* ATCC13032.

^b^ 98% gene similarity to *dtsR1* gene encoding detergent sensitivity rescuer in *C. glutamicum* ATCC13032.

^c^ 99% gene similarity to *dtsR2* gene encoding detergent sensitivity rescuer in *C. glutamicum* ATCC13032.

**Table S2.** Compositions of different lignocellulose biomass.

|  | Cellulose (%) | Hemicellulose (%) | Lignin (%) | Ash (%) |
| --- | --- | --- | --- | --- |
| Corn stover | 34.20 ± 0.24 | 31.50 ± 0.31 | 8.10 ± 0.09 | 5.24 ± 0.00 |
| Wheat straw | 41.25 ± 0.30 | 12.66 ± 0.20 | 10.24 ± 0.19 | 0.78 ± 0.00 |
| Rice straw | 37.67 ± 0.38 | 27.61 ± 0.47 | 2.58 ± 0.00 | 5.52 ± 0.01 |
| Sugarcane bagasse | 45.84 ± 0.33 | 31.64 ± 0.56 | 7.86 ± 0.00 | 1.62 ± 0.00 |
| *Phragmites communis* reeds | 42.47 ± 0.01 | 26.79 ± 0.03 | 7.28 ± 0.00 | 4.56 ± 0.00 |
| Poplar tree sawdust | 53.90 ± 2.47 | 16.82 ± 0.32 | 18.86 ± 0.02 | 0.23 ± 0.00 |

The composition of the raw biomass was determined using the two step sulfuric acid hydrolysis method according to the National Renewable Energy Laboratory (NREL) protocols [1, 2]. Standard deviations were derived from at least two independent determinations.

**Table S3.** Concentrations of glucose, xylose and inhibitor compounds in different corn stover hydrolysate (CSH).

| CSH | Glucose  (g/L) | Xylose (g/L) | Acetic acid  (g/L) | Furfural  (g/L) | HMF  (g/L) | Vanillin  (mg/L) | HBA  (mg/L) | Syringaldehyde  (mg/L) |
| --- | --- | --- | --- | --- | --- | --- | --- | --- |
| non-detoxified CSH | 63.2 ± 1.6 | 19.5 ± 1.2 | 3.7 ± 0.7 | 0.8 ± 0.1 | 0.5 ± 0.0 | 500 ± 77 | 10 ± 2 | 300 ± 78 |
| CSH at 15% solids content | 63.9 ± 3.6 | 27.4 ± 2.2 | 3.3 ± 0.1 | 0 ± 0 | 0 ± 0 | 20 ± 1 | 2 ± 0 | 60 ± 10 |
| CSH at 20% solids content | 90.4 ± 3.4 | 17.4 ± 3.7 | 1.4 ± 0.4 | 0 ± 0 | 0 ± 0 | 8 ± 1 | 3 ± 1 | 43 ± 5 |
| CSH at 25% solids content | 109.1 ± 1.8 | 26.7 ± 1.3 | 8.3 ± 0.6 | 0 ± 0 | 0 ± 0 | 13 ± 3 | 4 ± 0 | 200 ± 3 |

Non-detoxified CSH was prepared from 15% (w/w) of the freshly pretreated corn stover. CSH at 15%, 20% and 25% solids content (w/w) were prepared from 15% (w/w), 20% (w/w) and 25% (w/w) of the pretreated and biodetoxified corn stover. Standard deviations were derived from at least two independent determinations.

**Table S4.** Primers used in real-time quantitative PCR (RT-qPCR) assay.

| **Genes** | **Gene locus** | **Forward primer sequence (5'-3')** | **Reverse primer sequence (5'-3')** |
| --- | --- | --- | --- |
| *16S rRNA* | CGS9114_RS11955 | GCCCCTTATGTCCAGGGCTT | GGTCGAGTTGCAGACCCCAA |
| *ufaA* | CGS9114_RS06985 | ACTTTGCGGGCCATTACCTAAA | GCGGTAGACGGCATCAAAGC |
| *pgsA1* | CGS9114_RS06020 | AGCCTTGTTGGTTTGGGGCT | TGACCAAGCGCTGCACAACA |
| *pima* | CGS9114_RS07945 | GATGCGCAGTTGCTCATTGT | GGCAAGCGTGTTGATCATGT |
| *dacB* | CGS9114_RS00725 | ACCAGAACCCACCGACAACCT | CCAGGCTAGTGCGGCGATTA |
| *dac* | CGS9114_RS13375 | TACGCCTCGTCTGCTTGATG | CACCCGAGAGTCCGACGTAG |
| *ftsI* | CGS9114_RS11420 | ACTGCAGCCACCGGTTCAGTT | CGCTGCACATTGGCAAGGAC |
| *ftsI* | CGS9114_RS07835 | GTGTGGGACAGTCAACGGGT | CGACATACCCTGACCGATGG |
| *odhA* | CGS9114_RS03450 | GTCATGCTGGTCTCCGGCA | CCTCGGAGATGCGGTTGAAC |
| *sucB* | CGS9114_RS08045 | CCTTCACCATCACCAACATTGG | CGGTGATGACAACTGGACGC |
| *lpdA* | CGS9114_RS13335 | TTTGGCTACTAACCCACGCG | CACCACGACACCACCGATG |
| *lpd* | CGS9114_RS04910 | CTGCTCGGTGGACACCTCGTT | TTGGGTGAATGTGAACGCTGC |
| *gdh* | CGS9114_RS12025 | AACCGTGGCATCACTGTGGT | GCAGTCTCTGCACGGAATGC |
| *gdh* | CGS9114_RS07420 | CCTGAGGCTGTTGAGGTCTTC | CGAAGCGTTCTGCTGCAT |
| *yggb* | CGS9114_RS01440 | TGACCACCTCCGAAACTTCC | GTTGAGGTTTCCTGCTGCGT |
| *bioY* | CGS9114_RS11175 | GGTCTCGCGGGTCTGATCAC | GCACAAAGGCACCTTGAGCA |
| *bioM* | CGS9114_RS11180 | AATCACCGCACACCCTATCCG | CAGCAGGGTAGTGGGCTCATCA |
| *bioN* | CGS9114_RS11185 | AGCGGATGTTGCAGCCTTTTG | CACCGTGGCTAATTGCAGCG |
| *accBC* | CGS9114_RS13275 | ATCTCCGGACAGTTCGACTC | CCTCGACAACGTACTCTGCA |
| *accD1* | CGS9114_RS13235 | ACATCCCAATCGTCATGCTT | CCGTATGCGTAGAGCAGCTT |
| *accD2* | CGS9114_RS13240 | GTTCTGTTGGCATCGTGGCT | AGGAAGCCTGGAACGTCCAC |
| *accD3* | CGS9114_RS13505 | CCTGATCTACGCAGCCGA | CTGCACGCCTTGTCGTTC |
| *accD4* | CGS9114_RS12340 | TCCGTATCTGCGACGCCTAC | CACGGTGAATCAAACCGACC |
| *fasA* | CGS9114_RS13530 | CCACCGTCACTGGTGAAGAA | CGTCGTAGAGTTCGGTGTCG |

**References**

1. Sluiter A, Hames B, Ruiz R, Scarlata C, Sluiter J, Templeton D: Determination of sugars, byproducts, and degradation products in liquid fraction process samples. NREL/TP-510-42623, National Renewable Energy Laboratory, Golden, CO. 2008.

2. Sluiter A, Hames B, R. Ruiz CS, Sluiter J, Templeton D, Crocker D: Determination of Structural Carbohydrates and Lignin in Biomass. NREL/TP-510-42618 Laboratory Analytical Procedure (LAP), National Renewable Energy Laboratory Golden, CO. 2012.
